# Osimertinib Resistance: Molecular Mechanisms and Emerging Treatment Options

**DOI:** 10.3390/cancers15030841

**Published:** 2023-01-30

**Authors:** Georgia Gomatou, Nikolaos Syrigos, Elias Kotteas

**Affiliations:** Oncology Unit, Third Department of Medicine, “Sotiria” General Hospital for Diseases of the Chest, National and Kapodistrian University of Athens, 11527 Athens, Greece

**Keywords:** EGFR, NSCLC, osimertinib, targeted therapy, resistance

## Abstract

**Simple Summary:**

Osimertinib, a third-generation epidermal growth factor receptor (EGFR) tyrosine kinase inhibitor, is currently indicated as first-line therapy in patients with non-small-cell lung cancer (NSCLC) and sensitizing EGFR mutations; as second-line therapy in patients who present the resistance-associated mutation T790M after treatment with previous EGFR-TKIs; and as adjuvant therapy for patients with early stage, resected NSCLC, harboring EGFR mutations. Understanding the patterns of resistance, including both on-target and off-target mechanisms, as well as their therapeutic potential, represents an unmet need in thoracic oncology. Differential resistance mechanisms develop when osimertinib is administered in a first-line versus second-line setting. Standard therapeutic approaches after progression to osimertinib include other targeted therapies—when a targetable genetic alteration is detected—and cytotoxic chemotherapy with or without antiangiogenic and immunotherapeutic agents. Research should also be focused on the standardization of liquid biopsies in order to facilitate the monitoring of molecular alterations after progression to osimertinib.

**Abstract:**

The development of tyrosine kinase inhibitors (TKIs) targeting the mutant epidermal growth factor receptor (EGFR) protein initiated the success story of targeted therapies in non-small-cell lung cancer (NSCLC). Osimertinib, a third-generation EGFR-TKI, is currently indicated as first-line therapy in patients with NSCLC with sensitizing EGFR mutations, as second-line therapy in patients who present the resistance-associated mutation T790M after treatment with previous EGFR-TKIs, and as adjuvant therapy for patients with early stage resected NSCLC, harboring EGFR mutations. Despite durable responses in patients with advanced NSCLC, resistance to osimertinib, similar to other targeted therapies, inevitably develops. Understanding the mechanisms of resistance, including both EGFR-dependent and -independent molecular pathways, as well as their therapeutic potential, represents an unmet need in thoracic oncology. Interestingly, differential resistance mechanisms develop when osimertinib is administered in a first-line versus second-line setting, indicating the importance of selection pressure and clonal evolution of tumor cells. Standard therapeutic approaches after progression to osimertinib include other targeted therapies, when a targetable genetic alteration is detected, and cytotoxic chemotherapy with or without antiangiogenic and immunotherapeutic agents. Deciphering the when and how to use immunotherapeutic agents in EGFR-positive NSCLC is a current challenge in clinical lung cancer research. Emerging treatment options after progression to osimertinib involve combinations of different therapeutic approaches and novel EGFR-TKI inhibitors. Research should also be focused on the standardization of liquid biopsies in order to facilitate the monitoring of molecular alterations after progression to osimertinib.

## 1. Introduction

Activating mutations of the epidermal growth factor receptor (EGFR) gene are detected in 15–20% of patients with non-small-cell lung cancer (NSCLC), predominantly in patients with adenocarcinomas, without a smoking history, in women, and Asian patients [1]. The kinase domain of the protein EGFR is encoded by exons 18 to 24. The majority of NSCLC-related EGFR mutations are located in four exons, 18–21, clustering around the adenosine triphosphate (ATP)-binding pocket of the protein [1]. The most frequent mutations are a small deletion in exon 19 and the L858R mutation in exon 21 [2].

During the last two decades, the development and clinical application of EGFR tyrosine kinase inhibitors (EGFR-TKIs), namely the first-generation gefitinib, erlotinib, and icotinib; the second-generation afatinib and dacomitinib; and the third-generation osimertinib, have been associated with substantial responses and survival benefit in patients harboring EGFR mutations, paving the way for establishing a precision oncology approach regarding NSCLC [3]. A substituted amino acid, in particular, a change from threonine to methionine, at the “gatekeeper” 790 residue in exon20 of EGFR (p.Thr790Met or T790M mutation), was identified as a mechanism conferring resistance to first- and second-generation inhibitors in approximately 50% of the cases through the alteration of inhibitor specificity in the ATP-binding pocket of the protein [4].

**Figure 1:**
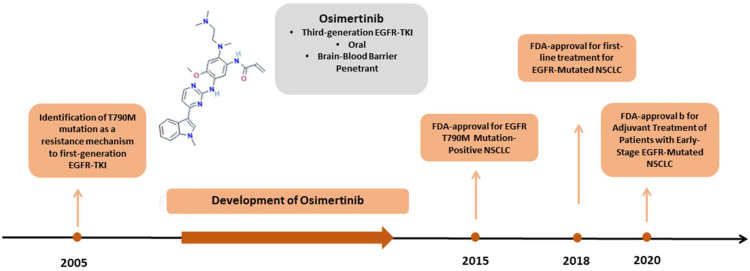
Timeline of development and approvals of osimertinib. Abbreviations: EGFR: epidermal growth factor receptor, TKI: tyrosine kinase inhibitor, FDA: Food and Drug Administration, NSCLC: non-small-cell lung cancer. Figure of osimertinib molecule: National Center for Biotechnology Information (2023). PubChem Compound Summary for CID 78357807, Osimertinib mesylate. Retrieved 24 January 2023 from https://pubchem.ncbi.nlm.nih.gov/compound/Osimertinib-mesylate.

Hence, osimertinib, an oral, irreversible, third-generation EGFR-TKI, was designed to target the T790M resistance-associated mutation as well as the L858R and exon19del activating mutations while sparing wild-type EGFR [5]. Osimertinib was initially approved as a second-line treatment in EGFR T790M-positive NSCLC, based on the results of the AURA trials [6]. In 2018, osimertinib was also approved as a first-line treatment of advanced EGFR-mutant NSCLC on the basis of the results from the FLAURA trial, reporting a progression-free survival (PFS) of 18.9 months and overall survival (OS) of 38.6 months in untreated patients harboring EGFR sensitizing mutations [7]. Of note, osimertinib penetrates the brain–blood barrier and presents substantial central nervous system activity, contrary to early generation EGFR-TKIs [7]. Finally, the ADAURA trial investigated the use of osimertinib as an adjuvant treatment in patients with resected NSCLC stage IB to IIIA harboring EGFR mutations, and the results demonstrated significantly longer disease-free survival compared with the placebo, resulting in the approval of the drug in the adjuvant setting [8] (Figure 1).

Despite the durable disease control, the majority of patients receiving osimertinib eventually develop disease progression, according to clinical and standardized radiological criteria. As the use of upfront osimertinib for advanced EGFR-mutant NSCLC is increasing, it is essential to understand the mechanisms of resistance to the drug in order to establish subsequent treatment. Because osimertinib is also used in the adjuvant setting, the patterns of resistance are relevant in the case of patients who relapse during the period of adjuvant treatment. The optimal management of patients after progression to osimertinib constitutes a therapeutic challenge [3]. The aim of this review is to summarize and critically discuss the current evidence on the mechanisms conferring resistance to osimertinib as well as the emerging therapeutic options in order to overcome resistance.

## 2. Mechanisms of Resistance

Osimertinib is a mono-anilino-pyrimidine compound that corresponds to the molecular formula C28H33N7O2 and possesses a molecular weight of 596 g/mol. Osimertinib irreversibly binds to mutant EGFR via its acrylamide group that forms a covalent bond with the Cys797 residue located in the ATP-binding site of mutant EGFR [9].

The mechanisms of resistance to osimertinib are generally divided into two categories: (a) those occurring as a genetic alteration at the EGFR gene (on-target mechanisms) and (b) those involving different genetic alterations and activation of other pathways (off-target mechanisms). Evidence from the clinical trials and real-world registries has revealed differential mechanisms when osimertinib is given as first-line or second-line treatment, underlying the discrepancies in the selection pressure and clonal evolution of tumor cells [10]. The relative frequencies of resistance mechanisms that have been reported from clinical trials and representative real-world cohorts are summarized in Table 1.

### 2.1. EGFR-Dependent (on-Target) Resistance Mechanisms

#### 2.1.1. T790M Loss

In 50–60% of patients treated with first- or second-generation EGFR-TKI, the T790M mutation develops, which results in the steric hindering of the drug’s binding to the mutant EGFR protein [4]. Osimertinib covalently binds to the cysteine-797 (C797) residue in the ATP-binding site of the protein, in addition to T790M, capable of overcoming the resistance mechanisms [9,19].

Analysis of circulating tumor (ct) DNA at the time of progression or treatment discontinuation revealed undetectable T790M in 49% of the patients in the AURA3 trial [11]. In most cases, the loss of T790M is associated with the development of alternative competing resistance mechanisms [12]. Oxnard et al. demonstrated that 63% of patients who received osimertinib for T790M-mutant NSCLC presented loss of T790M at the time of progression, which was frequently associated with the occurrence of histologic transdifferentiation, KRAS mutations, or gene fusions [12]. In the same study, the time to treatment discontinuation was shorter in patients with T790M loss (6.1 vs. 15.2 months, *p* = 0.01) [12]. Τhe loss of T790M was also associated with earlier resistance and poorer survival in other cohorts [16,20]. The loss of T790M and the presence of EGFR-activating mutations in plasma were associated with the shortest PFS in a genomic analysis of postprogression samples of patients who received second-line osimertinib (median 2.6 months, 95% CI 1.3, not reached) [20]. On the other hand, when osimertinib is used upfront, the T790M mutation does not usually exist either pre- or postprogression; therefore, given the increasing frontline application of osimertinib, the significance of T790M will possibly become less considerable [13].

#### 2.1.2. C797S Mutation

The emergence of another genetic alteration, a tertiary mutation in EGFR residue C797 in exon 20 in the ATP-binding site, represents a key mechanism that confers resistance to osimertinib [21]. Most frequently, serine substitutes cysteine at codon 797 (C797S) and another rare substitution involving glycine (C797G) have also been reported [21]. From a mechanistic point of view, osimertinib overcomes T790M resistance by forming a bond with the residue C797 in the ATP pocket; therefore, the protein modification caused by a mutation in the C797 residue hinders the covalent bond of osimertinib to the mutant EGFR [21].

Of note, C797S represents the most common EGFR-dependent mechanism of resistance to osimertinib taking into consideration all patients receiving osimertinib in any line of therapy; however, it predominantly occurs in the second-line setting [22]. More specifically, regarding the AURA3 trial, C797 mutations were detected in 15% of the patients at disease progression, while in the FLAURA trial, they were detected in 7% of the patients who progressed on osimertinib in the first line [11,13]. Additionally, the prevalence of C797 mutations is reported as 11% to 29% in real-world cohorts [14,15,17,18].

Importantly, in the case of patients who receive osimertinib as a second- or later-line therapy for T790-positive NSCLC, a triple-mutant scenario, with the genotype EGFRL858R/T790M/C797S or EGFRexon19del/T790M/C797S, may occur. Interestingly, the allelic context in which C797S is acquired matters and may predict the responsiveness to alternative treatments. In particular, if the C797S and T790M mutations are in different alleles (in trans), the tumor cells are resistant to osimertinib but may be sensitive to a combination of first- and third-generation TKIs. If the mutations exist in the same allele (in cis), then the currently approved EGFR-TKIs, alone or in combination, do not display antitumor activity [22]. Indeed, a preclinical study proved that cells harboring EGFR C797S in trans with T790M are sensitive to a combination of first- and third-generation EGFR TKIs. Case reports of patients who harbored the EGFR C797S mutation located in trans with T790M describe that the combination of a first-generation TKI with osimertinib was associated with a clinical and radiographic response [23,24]. In contrast, the emergence of the C797S mutation in cis with T790M, which is the most frequent scenario, precludes sensitivity to first- and second-generation TKIs [25].

Similarly, in the case of the upfront use of osimertinib, preclinical data demonstrate that upon occurrence of C797S the cancer cells retain the sensitivity to early generation inhibitors; however, it may be transient [26], and additional resistance mechanisms may coevolve with C797S [27] (Table 2).

#### 2.1.3. Rare EGFR Mutations

Rare EGFR mutations, including at positions L718, L792, G724, and G796, were also reported as mechanisms leading to tumor cells’ resistance to osimertinib [33].

Mutations at a residue of the P-loop (L718) in exon18, corresponding to the site of the protein that forms a hydrophobic sandwich with the phenyl aromatic ring of osimertinib, were shown to confer resistance to the drug [33]. The reported substitutions include L718Q and L718V. It has been shown that patients with NSCLC who harbor the combination of EGFR L858R/T790M/L718 mutations are resistant to first- and second-generation inhibitors, whereas tumors with L858R/L718, which may occur in the case of first-line osimertinib, seem to be sensitive to afatinib [28,29].

Additionally, the G724S mutation in exon 18 represents a mechanism of resistance that has been identified in cases of second-line therapy with osimertinib. Structural analysis and computational modeling indicate that the EGFR G724S mutation alters the glycine-rich loop of the protein, leading to the incompatibility of forming a covalent bond with osimertinib [30,34]. The authors demonstrated in vitro that exposure to afatinib led to a reduction in tumor growth of G724S-driven cells in cases of concomitant T790M loss [30]. The efficacy of afatinib in the case of T790M loss and emergence of G724S mutation was also outlined in a case report [31]. Interestingly, another study using simulations as well as in vitro experiments and patient genomic profiling suggested that the G724S mutation selectively confers resistance in the context of Ex19Del, while L858R/G724S cancer cells are sensitive to osimertinib [32].

Additionally, G796R and L792 are rare mutations that have been reported in NSCLC treated with osimertinib and might hamper the drug’s binding and ultimately reduce its activity [35,36]. Finally, the double-mutant cells T790M/M766Q were shown to be resistant to osimertinib but sensitive to neratinib and poziotinib (dual inhibitors of the human epidermal growth factor receptor 2 (HER2) and EGFR kinase) [37].

### 2.2. EGFR-Independent (off-Target Resistance Mechanisms)

#### 2.2.1. MET Amplification

The mesenchymal–epithelial transition (MET) oncogene encodes the receptor tyrosine kinase c-Met. The receptor is subsequently phosphorylated to the binding of its ligand, the hepatocyte growth factor (HGF), which is typically produced by mesenchymal cells. The phosphorylated receptor activates downstream signaling pathways, namely the phospho-inositide 3-kinase (PI3K) pathway, the Janus kinases/signal transducer and activator of transcription (JAK/STAT) pathway, and the mitogen-activated protein kinase (MAPK) pathway. The MET signaling cascades result in the recruitment of many signal transducers that regulate morphogenesis during tubule formation and are involved in cell mitogenesis [38].

MET amplification has been recognized as a pivotal EGFR-independent resistance mechanism, because it bypasses EGFR inhibition through the activation of its downstream pathways. It should be noted that MET amplification may occur as a focal amplification or as a result of chromosome 7 polysomy; the former occurs via mechanisms such as breakage–fusion–bridge, and the latter refers to multiple copies of chromosome 7 in tumor cells, secondary to factors such as chromosomal duplication [39]. Focal high-copy amplification of MET is an oncogenic driver event for cancer, whereas polysomy is typically not. The ratio of the mean MET gene copy per cell relative to chromosome 7 centromere (CEP7) is used to distinguish between polysomy and true amplification [39].

Currently, MET amplification is not consistently defined across different studies and assays. Typically, it is defined as the presence of a MET gene copy number of five or a MET/CEP7 ratio of two with the use of fluorescence in situ hybridization (FISH) [12]. Regarding next-generation sequencing (NGS), despite its advantages regarding the identification of multiple genetic alterations, its major disadvantage is that not all assays control for CEP7. So, when an increase in gene copy number of MET is detected, it may correspond to a polysomy rather than to a focal MET amplification [39]. It is therefore recommended to use FISH for the evaluation of MET amplification, especially in the case that the NGS assay does not clearly evaluate the gene copy number gain [40].

It has been estimated that 5–24% of disease progressions upon osimertinib use in any line of therapy are attributed to the development of MET amplification [11,12,13,14,15,17]. Interestingly, MET amplification is more common in the first-line setting, while C797S is more frequent in the second-line setting [41,42]. Regarding the second-line therapy, MET amplification was associated with other co-occurring alterations, such as loss of T790M, the emergence of C797S [11] and other genetic events, such as CDK6 and BRAF amplification [10]. Preclinical studies showed that EGFR-mutant, osimertinib-resistant, MET-amplified cell lines are sensitive to the combination of MET inhibitors with afatinib [43]; the relevant clinical investigation is discussed in another paragraph.

Of note, MET amplification should be distinguished from MET exon 14 (METex14) mutation, which occurs as a de novo alteration in 3% to 4% of lung adenocarcinomas [3]. More specifically, the MET receptor lacking exon 14 exhibits decreased protein turnover because of loss of the ubiquitination site encoded by exon 14, resulting in aberrant MET activation and oncogenesis. METex14 was identified as a mechanism of acquired resistance to erlotinib in a case report describing a patient who was later effectively treated with a combination of osimertinib and crizotinib (a TKI with dual anti-ALK and -MET activity) [44]; however, evidence of the role of METex14 as a resistance mechanism to osimertinib has not yet been reported.

#### 2.2.2. HER2 Amplification and HER2 Point Mutations

Another off-target mechanism enabling the bypass of EGFR inhibition through the activation of the downstream pathways is the amplification of the human epidermal growth factor 2 receptor (HER2), another tyrosine kinase receptor, which is encoded by the ERBB2 gene [45]. Based on data from the AURA and FLAURA clinical trials, HER2 amplification was detected in 5% of patients who developed resistance to second-line osimertinib and 2% of cases of first-line osimertinib. It seems that HER2 amplification is mutually exclusive with the T790M mutation [11,13].

Moreover, HER2 point mutations account for approximately 1.5% of acquired resistance to osimertinib [46]. The majority of HER2 mutations comprise insertions in exon 20. It was shown that such genetic alterations affect the kinase domain of HER2 [46]. Another rare HER2 genetic alteration was also implicated in resistance to osimertinib according to a preclinical study, which demonstrated that exon 16-skipping HER2 led to the formation of a splice variant of the protein (HER2D16 variant) via a Src-independent pathway [47]. In vitro, HER2D16-expressing cells were resistant to osimertinib, and combination therapy of osimertinib with a Src kinase inhibitor failed to reverse resistance. In contrast, the combination of osimertinib with the pan-HER small-molecule inhibitor afatinib synergistically suppressed cell proliferation and signaling in HER2D16 cells [47].

#### 2.2.3. HER3 Upregulation

HER3 is another member of the EGFR family, but without an active kinase domain. However, it heterodimerizes with other HER proteins, enabling its trans-phosphorylation, and it mediates downstream signaling mainly through the PI3K/protein kinase B (AKT) pathway. It was observed that upon in vitro and in vivo treatment with HER-family-TKIs, including osimertinib, HER3 may display reduced phosphatase activity and increased membrane expression, corresponding to a shift in its phosphorylation–dephosphorylation equilibrium and sustained activation of PI3K pathway [48].

U3-1402 is a novel antibody–drug conjugate (ADC) incorporating a HER3-antibody coupled with a topoisomerase I inhibitor (DXd). In preclinical models of EGFR-TKI resistant NSCLC, U3-1402 showed anticancer activity alone or in combination erlotinib [49]. Recently, another study revealed that osimertinib pretreatment led to overexpression of HER3-enhanced uptake of HER3-targeted therapy with U3-1402 in lung cancer cells, suggesting the combination of osimertinib and U3-1402, with the aim to delay the development of resistance [50]. The cotreatment with osimertinib and HER3 was further investigated in a preclinical study, which demonstrated that this drug combination led to enhanced immune antitumor toxicity via the activation of STING [51].

#### 2.2.4. RAS-RAF Pathway

The RAT sarcoma virus (RAS)/Raf murine sarcoma viral oncogene homolog B (RAF) pathway (also referred to as mitogen-activated protein kinase (MAPK) pathway) has a crucial role in cell growth, division, and differentiation [52].

Various genetic alterations of the MAPK pathway provide resistance to osimertinib [53]. In a preclinical model of EGFR-mutant cells exposed to several EGFR-TKIs including osimertinib, the results revealed a range of alterations in the MAPK pathway. Firstly, several NRAS mutations were detected, including a novel E63K mutation. Furthermore, the results demonstrated a gain of copy number of wild-type NRAS or wild-type KRAS [54]. Among the patients who progressed on osimertinib in the phase III clinical trials FLAURA and AURA3, mutations of NRAS and KRAS were detected in 3% and 1%, respectively [11,13]. BRAF mutations (primarily V600E) and BRAF amplifications and fusions have also been detected upon progression to first- or second-line osimertinib [13,14,15,55].

In a preclinical investigation, BRAF V600E-mutant, osimertinib-resistant cell lines were sensitive to a combination of a BRAF inhibitor (encorafenib) and osimertinib [56]. In a case report, the authors described that osimertinib combined with vemurafenib showed efficacy in a patient with BRAF V600E-mediated osimertinib resistance, who exhibited a clinical response for 13.4 months [57]. The concurrent combination of dabrafenib and trametinib plus osimertinib was also attempted in two patients with BRAF V600E NSCLC postprogression to osimertinib, but, due to toxicity, one patient ultimately received a reduced dose of dabrafenib and trametinib. Rebiopsy upon tumor progression revealed loss of BRAF V600E and emergence of EGFR C797S [58].

#### 2.2.5. PI3K Pathway

The PI3K/protein kinase B (also known as AKT)/mammalian target of rapamycin (mTOR) pathway, which is an important regulator of the cell cycle and proliferation, is dysregulated in 4–11% of patients who progress to osimertinib [12,14]. Most genetic alterations are found in PIK3CA (E454K, E542K, R88Q, N345K, and E418K), which encodes the p100α protein, the catalytic subunit of PI3K [59,60]. Another alteration commonly found in several cancers, the PTEN deletion, leads to overexpression of PI3K signaling and may contribute to osimertinib resistance [60]. Interestingly, histologic transformation of osimertinib-resistant adenocarcinomas to squamous or small-cell carcinomas, which is discussed in the next paragraph, is associated with acquired genomic alterations related to the PI3K/AKT/mTOR pathway, such as PTEN deletion mutations [61].

Contrary to other oncogenic driver mutations in NSCLC, which are generally mutually exclusive, PIK3CA mutations frequently coexist with other oncogenic gene mutations, which is attributed to the cooperative role of PIK3CA in mitogenic signaling during lung carcinogenesis [62,63].

#### 2.2.6. Oncogenic Fusions

Chromosomal rearrangements involving driver oncogenes, termed oncogenic fusions, have been identified not only as de novo oncogenic events but also as genetic alterations conferring resistance to osimertinib (4–7%) [11,12]. The involved oncogenes include anaplastic lymphoma kinase (ALK), BRAF, fibroblast growth factor receptor (FGFR), neurotrophic tyrosine receptor kinase (NTRK), rearranged during transfection (RET), and ROS proto-oncogene 1 (ROS1). Some of the specific rearrangements that have been reported are the following; SBTBN1-ALK, PLEKHA7-ALK, AGK-BRAF, PCBP2-BRAF, ESYT2-BRAF, BAIAP2L1-BRAF, FGFR3-TACC3, NTRK-TMP3, RET-ERC1, CCD&-RET, NCOA4-RET, and GOPC-ROS1 [11,12,13,64].

Oncogenic fusions are rare but potentially targetable acquired resistance mechanisms. Several case reports have been published presenting patients receiving combination therapies of osimertinib with or without another targeted therapy specific for the acquired fusion, e.g., crizotinib, selpercatinib, and alectinib, and achieving durable responses [64,65,66,67,68,69]. Interestingly, data on the tumor cells’ evolution dynamics of EGFR-mutant NSCLC revealed that fusions do not commonly emerge as single alterations; rather, they frequently co-occur with additional on or off-target” mechanisms of resistance [63].

#### 2.2.7. Cell Cycle Aberrations

The operation of the cell cycle, chiefly regulated by cyclins and cyclin-dependent kinases (CDKs), is often altered in cancer, leading to sustained proliferation of tumor cells [70]. Genetic events affecting the cell-cycle-related genes are detected in 10–12% of patients upon progression to osimertinib. The occurrence of those alterations has been associated with a shorter median PFS [11,13,59]. The cell-cycle-related alterations mainly include but are not limited to (a) gene amplifications, mainly of the genes encoding for cyclin D1, D2, E1, and CDK 4 and 6; and (b) deletion mutations in the CDK inhibitor 2A gene (CDKN2A), which encodes for the natural inhibitors of the pathway [11,13].

CDK4 and 6 phosphorylate the retinoblastoma protein (Rb), which is a major determinant of sustained cell proliferation in cancer, such as in the case of osimertinib-resistant cancer cells independent of the underlying mechanisms. So, La Monica et al. examined the role of phosphorylated Rb as a biomarker and/or as a target in NSCLC-osimertinibin-resistant cells and showed that Rb phosphorylation was maintained in the majority of NSCLC cell lines with resistance to osimertinib [71]. According to preclinical studies, combining osimertinib with the CDK inhibitors abemaciclib or palbociclib leads to a decline of Rb phosphorylation and hinders the cell proliferation through an arrest of the resistant cells in the G1 phase [72]. Currently, an ongoing phase II clinical trial was designed to assess the safety and efficacy of abemaciclib, with or without osimertinib, in NSCLC after osimertinib resistance (ClinicalTrials.gov, NCT04545710).

#### 2.2.8. Histologic Transformation

Remarkably, 2–15% of patients who progress on osimertinib treatment in any line develop histologic transformation of their tumor from adenocarcinoma to squamous cell carcinoma or small-cell lung cancer (SCLC) [12,15,16,17]. In a recent study, histologic transformation was identified in 15% of first-line osimertinib cases and 14% of later-line cases [73]. The initial EGFR sensitizing mutation are retained both in squamous and small-cell transformation [74,75].

The presence of SCLC transformation has been associated with the loss of both RB1 and TP53 when genotyping the tumors [76,77]. The presence of apolipoprotein B mRNA editing enzyme was also linked to a histologic shift of the tumor [76]. Therefore, whenever concurrent RB1 and TP53 alterations are detected, potentially with the use of liquid biopsy, a tissue biopsy is absolutely required in order to specify the histologic type. Additionally, it was shown that the patients who harbor EGFR/RB1/TP53 mutations at baseline, who represent 5% of patients with EGFR-mutant NSCLC, represent a distinct subgroup of patients at high risk of histologic transformation but also with poorer outcome irrespective of the transformation itself [77].

There are no specific indications regarding the therapeutic management of EGFR-mutant NSCLC with transformed histology, which is generally associated with a poor prognosis, mainly attributed to its aggressive biology [3]. Histology-driven traditional chemotherapeutic regimens could be given in this patient subgroup, but with limited efficacy [78]. Currently, the combination of a PARP inhibitor (niraparib) and anti-PDL1 durvalumab is being tested in EGFR-mutant NSCLC that has transformed to a SCLC phenotype in a phase II clinical trial (NCT04538378).

#### 2.2.9. Epithelial to Mesenchymal Transition (EMT)

Epithelial–mesenchymal transition (EMT), a process during which the epithelial cells experience the loss of their polarity and adhesion capacities and acquire of a mesenchymal phenotype enabling cellular migration, has been described as a mechanism contributing to osimertinib resistance [79]. A preclinical model of osimertinib-resistant cells showed that the emergence of EMT resulted from osimertinib-induced upregulation of transforming growth factor beta 2 (TGFβ2) that activated SMAD2 protein [79]. In the same study, the resistant cell lines were highly dependent on the NF-κB pathway for survival, and treatment with the NF-κB pathway inhibitor BAY 11–7082 or genetic silence of p65, one of the components of NF-κΒ, yielded a cytotoxic effect [79].

Because EMT collectively refers to a differential phenotype rather than a single alteration, it is of utmost importance to characterize the specific implicated pathways in order to harness them therapeutically. Well-defined EMT-associated alterations involve the decrease in E-cadherin, a contributor of the adherens junctions, and the overexpression of the mesenchymal biomarker, vimentin, as well as of Hakai protein, a degrader of E-cadherin [80,81]. The regulators of E-cadherin expression, namely the transcription factors Zeb, Snail, Slug, and twist, are highly considered potential therapeutic targets [82]. In a preclinical study, TWIST1 overexpression resulted in erlotinib and osimertinib resistance in EGFR-mutant NSCLC cells; conversely, genetic and pharmacological inhibition of TWIST1 increased the sensitivity to EGFR TKIs [83].

**Figure 2:**
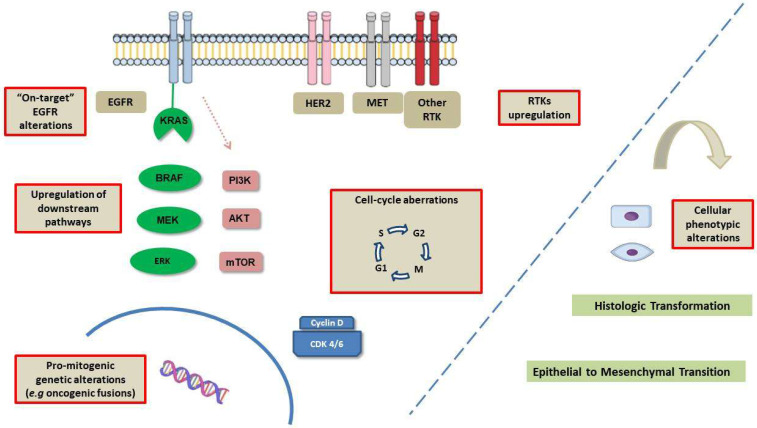
Overview of mechanisms of resistance to osimertinib. In addition to EGFR-dependent (on-target) alterations, different mechanisms of resistance occur at several levels within the cell. These include upregulation of other receptor tyrosine kinases, upregulation of downstream pathways, oncogenic fusions encoding for mutant, promitogenic proteins, and alterations affecting the regulation of the cell cycle. Additionally, histologic transformation to squamous or small-cell lung cancer may develop that confers resistance to EGFR inhibition. Finally, epithelial-to-mesenchymal transition contributes to developing resistance to osimertinib. Abbreviations: EGFR: epidermal growth factor receptor, RAS: rat sarcoma viral oncogene homolog, BRAF: v-Raf murine sarcoma viral oncogene homolog B, PI3K: phosphatidylinositol 3 kinase, AKT: protein kinase B, mTOR: mammalian target of rapamycin, MEK: mitogen-activated protein kinase kinase ERK: extracellular signal-regulated kinase, RTKs: receptor tyrosine kinases.

An overview of mechanisms of resistance to osimertinib is illustrated in Figure 2.

## 3. Management of Osimertinib-Resistant NSCLC

Optimizing the next step after progression of EGFR-mutant NSCLC while on osimertinib is an ongoing challenge and this issue is not clearly defined in therapeutic algorithms [84]. Performing a rebiopsy in order to molecularly characterize the tumor is recommended; however, a tissue biopsy is sometimes difficult to obtain due to the location of the tumor masses or the patient’s physical condition or it may be time-consuming. The use of liquid biopsy is recommended as long as the limitations of this technology are taken into consideration [85]. The next therapeutic step should be tailored to each patient and may depend on the detection of specific resistance-associated genetic alterations. Several therapeutic options beyond progression to osimertinib are discussed below.

### 3.1. Targeted Therapy for EGFR-Dependent Alterations

As discussed above, in the case of NSCLC with loss of T790M and acquired tertiary mutations (e.g., C797S), the use of an early generation EGFR-TKI appears a feasible therapeutic option after progression on osimertinib. Similarly, if T790M mutation coexists with tertiary mutations but on different chromosomes (in trans), it is presumed that the cancer cells will remain sensitive to treatment with a combination of osimertinib and a first-generation inhibitor. A number of case reports have suggested using early generation EGFR-TKIs as monotherapy or in combination with osimertinib in those scenarios [26,27,86,87,88]. However, this therapeutic approach has not been evaluated in randomized clinical trials. Additionally, preclinical evidence suggests that the sensitivity of cancer cells to early generation EGFR-TKIs may be transient [26].

The development of targeted therapy for C797S-mediated resistance to osimertinib represents an unmet need and is currently actively explored [45]. To this end, fourth-generation EGFR-TKIs, such as LS-106, BLU-945, BLU-701, EAI045, and JBJ-09-063, have been specifically designed to evade the resistance caused by the EGFR C797S mutation.

The fourth-generation allosteric EGFR inhibitors bind to a different site than the existing ATP-competitive EGFR TKIs, modifying the space configuration of mutant EGFR and hindering its binding to EGFR ligands, therefore blocking its own phosphorylation and its downstream signals. All agents mentioned above have shown preclinical in vitro and in vivo antitumor efficacy in models of NSCLC harboring C797S and common activating EGFR mutations, with or without T790M [89,90,91,92].

Regarding clinical investigation, SYMPHONY (NCT04862780) is a phase I/II clinical trial that is currently recruiting patients with EGFR-mutant NSCLC after failure of third-generation EGFR-TKIs, aiming to assess the safety, tolerability, and anticancer activity of BLU-945 with or without osimertinib. Similarly, HARMONY (NCT05153408) is a phase I/II clinical trial evaluating the safety, tolerability, and anticancer activity of BLU-701 alone or in combination with osimertinib or platinum-based chemotherapy in EGFR-mutant NSCLC. Finally, BDTX-1535 is another inhibitor that is currently being explored in a first-in human clinical trial for patients with glioblastoma and NSCLC harboring sensitive EGFR alterations and who have disease progression following standard of care (NCT05256290).

Furthermore, in addition to the use of EGFR-TKIs, it was reported that amivantamab, which acts as a bispecific anti-EGFR and anti-MET inhibitor, showed anticancer activity in a preclinical model of EGFR-mutant NSCLC cells with EGFR-TKIs resistance. It also presented clinical efficacy in patients with C797S mutation and concomitant MET amplification [93,94]. Additionally, the combination of an ALK-TKI (brigatinib) plus an anti-EGFR monoclonal antibody (cetuximab) was efficacious according to a retrospective study that included patients with T790M/cisC797S mutations resistant to osimertinib [94].

### 3.2. Targeted Therapy for EGFR-Independent Alterations

#### 3.2.1. MET Amplification

Preclinical data and preliminary clinical evidence support that a combination regimen of osimertinib and MET inhibitors is a reasonable therapeutic strategy to overcome the osimertinib resistance attributed to MET amplification. More specifically, durable responses associated with the combination treatment of crizotinib with osimertinib have been presented in case reports [95,96]. The efficacy of crizotinib and osimertinib was also analyzed in a retrospective cohort of patients with NSCLC, MET amplification, and resistance to prior EGFR-TKI [93]. The results showed that the objective response rate (ORR) was 100%, and the median PFS was 6.2 months [97].

The phase 1b TATTON study enrolled patients with advanced EGFR-mutant NSCLC who progressed on a prior EGFR-TKI in order to assess the safety and tolerability of osimertinib combined with selumetinib (MEK1/2 inhibitor), savolitinib (MET inhibitor), or durvalumab (anti-PD-L1 monoclonal antibody) [98]. Based on the outcome, the combination of osimertinib 80 mg with selumetinib or savolitinib at identified tolerable, active doses is possible, whereas the combination of osimertinib with durvalumab was not feasible due to the high incidence of toxicity (interstitial lung disease) [98]. Subsequently, the combination of osimertinib with savolitinib was assessed in two global expansion cohorts of the TATTON study. The interim analysis revealed that osimertinib plus savolitinib had an acceptable safety profile and observed antitumor activity; therefore, the final results are eagerly anticipated [99].

Additional clinical trials (NCT03778229, NCT05015608, and NCT03944772) are currently exploring the safety and efficacy of osimertinib combined with savolitinib. The combination of osimertinib with other MET inhibitors (capmatinib and tepotinib) is also under investigation in clinical trials (NCT04816214 and NCT03940703). Finally, telisotuzumab vedotin (Teliso-V), an ADC composed of a Met antibody (ABT-700) and a microtubule inhibitor (monomethyl auristatin E), is being explored in the LUMINOSITY trial (NCT03539536) that is currently recruiting pretreated patients with EGFR-mutant NSCLC and immunohistochemically positive c-MET.

#### 3.2.2. HER2 Amplification

Preclinical studies of HER2 amplification-mediated osimertinib-resistant models showed sensitivity to osimertinib in combination with the anti-HER2 ADC trastuzumab emtansine (TDM1). Li et al. reported clinical activity of TDM1 in patients with NSCLC and HER2 amplification who progressed on previous EGFR-TKIs [100]. Moreover, the authors described that when a patient with NSCLC developed resistance to TDM1, the switching of ADC from TDM1 to fam-trastuzumab deruxtecan (T-DXd) resulted in a partial response that strikingly lasted for one year [100]. The combination of osimertinib and TDM1 was investigated in a phase I/II trial (TRAEMOS) in patients with HER2 amplification-mediated EGFR-TKI resistance. However, the trial was terminated due to insufficient effectiveness (NCT03784599).

DESTINYLung01 was a phase II clinical trial that evaluated the efficacy and safety of T-DXd in patients with HER2-overexpressing or HER2-mutant NSCLC. According to a separate interim analysis of the trial, treatment with T-DXd resulted in a 24.5% ORR in HER2-overexpressed, immunohistochemistry (IHC) 2+ or 3+, but HER2 mutation-negative NSCLC, a status that may partially correspond to HER2 amplification. It must be noted that the initial design of this two-cohort phase II trial included a patients group with HER2 overexpression, classified with an immunohistochemical score of 3+ or 2+, and another group with HER2 mutations [101].

#### 3.2.3. Osimertinib in Combination with Inhibitors of Oncogenic Genetic Alterations

As aforementioned, several case reports propose a regimen of the combination of osimertinib plus a relevant inhibitor when a targetable oncogenic mutation or fusion is detected [60,102,103,104]. In addition to the case reports, the combination of osimertinib with a RET inhibitor (BLU-667) was supported by the results of an in vitro study, which suggested that the mechanisms of resensitization of osimertinib are mediated by the suppression of both extracellular -signal-regulated kinase (ERK) and AKT phosphorylation [65].

Combining osimertinib with another inhibitor targeting the corresponding oncogenic alteration appears to be a plausible therapeutic strategy; however, it should be noted that the tolerability of those combinations has not been assessed in clinical trials. For example, in a patient with BRAF V600E, osimertinib-resistant NSCLC, serious toxicity led to a dose reduction of dabrafenib plus trametinib in combination with the ordinary dose of osimertinib [59]. Therefore, not all such combination strategies are tolerable and feasible, which must attract the attention of physicians.

### 3.3. Beyond Targeted Therapy

#### 3.3.1. Chemotherapy

It must be noted that 30–50% of mechanisms resulting in resistance to osimertinib remain unknown, and, often, no targetable alteration is detected, despite performing a rebiopsy. Hence, targeted therapy may not be feasible, while platinum-based doublet chemotherapy remains a plausible option. Interestingly, the results from a retrospective cohort of patients with advanced NSCLC who progressed on osimertinib therapy showed that chemotherapy was correlated with a tendency toward longer survival compared with a nonchemotherapy regimen. The median OS was 25.0 versus 11.8 months, while median PFS was not significantly different between the two groups [105].

Resistance to osimertinib is attributed to a shift to a SCLC phenotype in 2–15% of cases [14,15,17,42]. Derived from primary SCLC, a platinum–etoposide combination therapy is routinely used to treat patients with SCLC transformation after osimertinib treatment. A retrospective study demonstrated that platinum–etoposide yielded a clinical response rate of 54%, a median PFS of 3.4 months, and a median OS of 10.9 months in patients with SCLC-transformed lung cancer. In the same study, a group of patients who received immune checkpoint inhibitors was analyzed, and the results showed that immunotherapy was largely inefficacious [74]. Currently, data do not support the continuation of osimertinib when commencing chemotherapy after disease progression on first-line therapy with osimertinib [106].

#### 3.3.2. Immunotherapy

Numerous clinical trials have demonstrated the clinical benefit of immune checkpoint inhibitors (ICIs) in the management of NSCLC; however, ICI monotherapy exhibits limited benefit in patients harboring EGFR mutations. A systematic review showed that ICIs were associated with prolonged OS (HR, 0.69; *p* < 0.001) in individuals harboring wild-type EGFR but not in the EGFR mutation subgroup (HR, 1.11; *p* = 0.54) compared with docetaxel [107]. Consistent with the above, the results from the IMMUNOTARGET registry, which included patients with oncogene-driven NSCLC who received immunotherapy, showed that ICI monotherapy yielded a short median PFS of 2.1 months and a low ORR of 12% among patients with NSCLC harboring EGFR alterations [108]. Of note, in patients receiving monotherapy with ICI and harboring an oncogene “driver” alteration, there is an increased risk of hyperprogressive disease, a severe complication that is related to worse survival [109].

#### 3.3.3. Combination of Chemotherapy and Antiangiogenic Therapy

The addition of vascular endothelial growth factor (VEGF) inhibitors to EGFR-TKIs in the first-line treatment of patients with EGFR-mutant NSCLC has been investigated; differential results have been revealed regarding the survival benefit between the combination of erlotinib plus bevacizumab [110] and osimertinib plus bevacizumab [111], a phenomenon that may reflect the tumor cell heterogeneity caused by different EGFR-TKIs. Nevertheless, the addition of an antiangiogenic agent to EGFR-TKI after progression to osimertinib has not been evaluated.

#### 3.3.4. Combination of Chemotherapy/Immunotherapy/Antiangiogenic Therapy

Recently, the combination of chemotherapy, immunotherapy, and antiangiogenic therapy has been examined in patients with EGFR-mutant NSCLC. Initially, the Impower130 study showed that no survival benefit was observed in the EGFR-mutant subgroup who received atezolizumab and carboplatin/nab-paclitaxel compared with those who received chemotherapy alone; however, the IMpower150 trial demonstrated that the addition of atezolizumab and bevacizumab to carboplatin/paclitaxel significantly improved PFS and OS in EGFR-mutated NSCLC patients, including those with previous TKI failures [112,113]. Similarly, another clinical trial reported that combination treatment with atezolizumab, bevacizumab, and carboplatin/pemetrexed was associated with promising efficacy in metastatic EGFR-mutant NSCLC patients after EGFR-TKI failure [114]. Another combination, that of sintilimab (anti-PD-1 inhibitor) plus bevacizumab and platinum–pemetrexed, in patients who progressed on EGFR-TKIs resulted in the prolongation of PFS compared with patients receiving chemotherapy alone [115]. Therefore, the results of those trials suggest a synergistic effect of ICIs and antiangiogenic drugs on chemotherapy for the treatment of NSCLC patients in EGFR-mutated settings.

## 4. Discussion

Osimertinib currently represents the standard of care for the upfront management of patients with advanced NSCLC harboring common activating mutations. Due to its increasing use in front-line settings, deciphering its associated resistance patterns is becoming more and more relevant. The literature review revealed a heterogeneous set of resistance mechanisms, both EGFR-dependent and -independent, and, to some extent, different from those that have been identified with the use of osimertinib in later-line settings. Taking into consideration the variation in the resistance patterns, it becomes evident that a tailored approach should be followed for each patient, mainly depending on the molecular profile of the progressed tumor.

In this context, performing a rebiopsy upon progression to osimertinib is of crucial importance for defining further management. Whenever a tissue biopsy is feasible, it should be pursued. Otherwise, tumor genotyping with a liquid biopsy is recommended, as long as the limitations of such an approach are taken into consideration [85]. More specifically, ctDNA is unsuitable for histological transformation evaluation and gene fusion acquisition screening, which typically requires the RNA NGS evaluation of a tissue sample [17]. Of note, liquid biopsy for tumor genotyping is an ongoing research area encompassing different technologies in addition to ctDNA; a broader potential may be available in the future. However, attention should be paid to the validation and standardization of such approaches in order to reliably drive therapeutic decisions.

Intriguingly, EGFR-mutant NSCLC increasingly becomes a disease entity requiring a distinct approach from the early to the advanced stage. Of note, osimertinib has also been approved for adjuvant treatment of resected NSCLC, which reveals additional questions regarding patients who may relapse during adjuvant treatment or after the completion of the adjuvant treatment. Data on patterns of resistance and optimal management are still scarce for such cases. Recently, the European Society of Medical Oncology (ESMO) published consensus statements for the management of EGFR-mutant NSCLC, highlighting the requirement for a distinct approach from diagnosis to management and from early to advanced stage [116].

## 5. Conclusions

Despite the durable responses of osimertinib, especially in the front-line setting, the majority of patients develop resistance. The mechanisms of resistance include EGFR-dependent alterations, predominantly the acquisition of C797S mutation, which hinders the binding of the drug; and EFGR-independent mechanisms, including MET and HER2 amplifications, KRAS/BRAF/PIK3CA mutations, oncogenic fusions, and histologic transformation. A rebiopsy upon progression is highly recommended in order to guide the next therapeutic step. Fourth-generation EGFR-TKIs are under clinical investigation, and the results of clinical trials are anticipated. Other therapeutic options include other targeted therapies whenever feasible, and the combination of chemotherapy with immunotherapy and antiangiogenic therapy, or cytotoxic chemotherapy alone. Future research should be focused on the expansion and validation of liquid biopsies to optimize the monitoring of the disease.

## Figures and Tables

**Table 1:** Relative frequencies of resistance mechanisms that have been reported from clinical trials and representative real-world cohorts.

Author (Year)	Number of Patients	Line of Therapy	EGFR-Dependent Mechanisms (On-Target)	EGFR-Independent Mechanisms (Off-Target)	[Ref]
Papadimitrakopoulou et al. (2018)	73	2nd line	T790M loss (49%) C797 mutations (15%; 10 patients with C797S, 1 patient with C797G)	MET amplification (19%) HER2 amplification (5%) PIK3CA amplification (4%) BRAFmut (V600E) (4%) KRAS mutation (1%) PIK3CA mut (E545K) (1%) FGFR/RET/NTRK fusions (4%)	[11]
Oxnard et al. (2018)	41	2nd line	T790Mloss (63%) C797S (22%)	SCLC transformation (15%) MET amplification (10%) BRAF mutation (5%) PIK3CA mutation (5%) KRAS mutation (2%) CCDC6-RET fusion (2%) FGFR fusion (2%) BRAF fusion (2%)	[12]
Ramalingam et al. (2018)	91	1st line	C797S (7%)	MET amplification (15%) HER2 ampl, PIK3CAmut, RAS mut (2–7%)	[13]
Enrico et al. (2019)	31	Any	C797S (29%) L817Q (6%) EGFR amplification (3%)	Oncogenic fusions (RET, MET, BRAF, ALK, FGFR3, and NTRK1) 16% BRAF mutation (V600E) 6% (co-existing with C797S) MET amplification (3%) HER2 amplification (3%) KRAS mutation (3%) PIK3CA mutation (3%)	[14]
Mehlman et al. (2019)	73	Any	T790M loss (68%) C797S (12%)	MET amplification (11%) Histologic transformation (9% of patients who underwent a tissue biopsy) HER2 amplification (3%) BRAF mutation (V600E) (1%)	[15]
Lee et al. (2021)	34	2nd line	T790Mloss (65%) C797S (12%)	SCLC transformation (9%) Squamous cell carcinoma transformation (5%) MET amplification (15%)	[16]
Akli et al. (2022)	27	1st line	C797S (11%)	MET amplification (15%) HER2 amplification (4%) SCLC transformation RET fusion (4%)	[17]
Nie et al. (2022)	21	1st line	C797S (24%) L718Q (5%) EGFR amplification (1%)	MET amplification (29%) HER2 amplification (10%) PTEN loss (5%) PIK3CA mutation (5%)	[18]

**Table 2:** EGFR mutations conferring resistance to osimertinib. Specific mutations/combination of mutations are sensitive to early generation EGFR-TKIs (in certain cases, in an allele-specific pattern).

EGFR Mutations	3rd-Generation EGFR-TKI Alone	1st/2nd Generation with or without 3rd Generation EGFR-TKI	[Ref]
del_19 or L858R/T790M/C797S (in cis)	Resistance	Resistance	[25]
del_19 or L858R/T790M/C797S (in trans)	Resistance	Sensitivity (1st-gen plus 3rd-gen EGFR-TKIs)	[22,23,24]
del_19 or L858R/C797S	Resistance	Sensitivity * (1st-gen plus 3rd-gen EGFR-TKIs)	[26]
L858R/T790M/L718	Resistance	Resistance	[28]
L858R/L718	Resistance	Sensitivity (2nd-gen EGFR-TKI)	[29]
del_19/G724S	Resistance	Sensitivity (2nd-gen EGFR-TKI)	[30,31]
L858R/G724S	Sensitivity **	Sensitivity	[32]

* Preclinical data show that sensitivity may be transient in such cases. ** Based on preclinical data and computations analysis.
